# The Gut–Brain Axis in Schizophrenia: The Implications of the Gut Microbiome and SCFA Production

**DOI:** 10.3390/nu15204391

**Published:** 2023-10-16

**Authors:** Songhyun Ju, Yoonhwa Shin, Sunhee Han, Juhui Kwon, Tae Gyu Choi, Insug Kang, Sung Soo Kim

**Affiliations:** 1 Department of Biomedical Science, Graduate School, Kyung Hee University, Seoul 02447, Republic of Korea; thdgus8543@khu.ac.kr (S.J.); jac03032@khu.ac.kr (Y.S.); sunheehan@khu.ac.kr (S.H.); kwonjh@khu.ac.kr (J.K.); 2 Department of Biochemistry and Molecular Biology, School of Medicine, Kyung Hee University, Seoul 02447, Republic of Korea; chtag@khu.ac.kr; 3 Biomedical Science Institute, Kyung Hee University, Seoul 02447, Republic of Korea

**Keywords:** schizophrenia, gut microbiota, gut–brain axis, blood–brain barrier, short-chain fatty acids

## Abstract

Schizophrenia, a severe mental illness affecting about 1% of the population, manifests during young adulthood, leading to abnormal mental function and behavior. Its multifactorial etiology involves genetic factors, experiences of adversity, infection, and gene–environment interactions. Emerging research indicates that maternal infection or stress during pregnancy may also increase schizophrenia risk in offspring. Recent research on the gut–brain axis highlights the gut microbiome’s potential influence on central nervous system (CNS) function and mental health, including schizophrenia. The gut microbiota, located in the digestive system, has a significant role to play in human physiology, affecting immune system development, vitamin synthesis, and protection against pathogenic bacteria. Disruptions to the gut microbiota, caused by diet, medication use, environmental pollutants, and stress, may lead to imbalances with far-reaching effects on CNS function and mental health. Of interest are short-chain fatty acids (SCFAs), metabolic byproducts produced by gut microbes during fermentation. SCFAs can cross the blood–brain barrier, influencing CNS activity, including microglia and cytokine modulation. The dysregulation of neurotransmitters produced by gut microbes may contribute to CNS disorders, including schizophrenia. This review explores the potential relationship between SCFAs, the gut microbiome, and schizophrenia. Our aim is to deepen the understanding of the gut–brain axis in schizophrenia and to elucidate its implications for future research and therapeutic approaches.

## 1. Introduction

**Figure 1:**
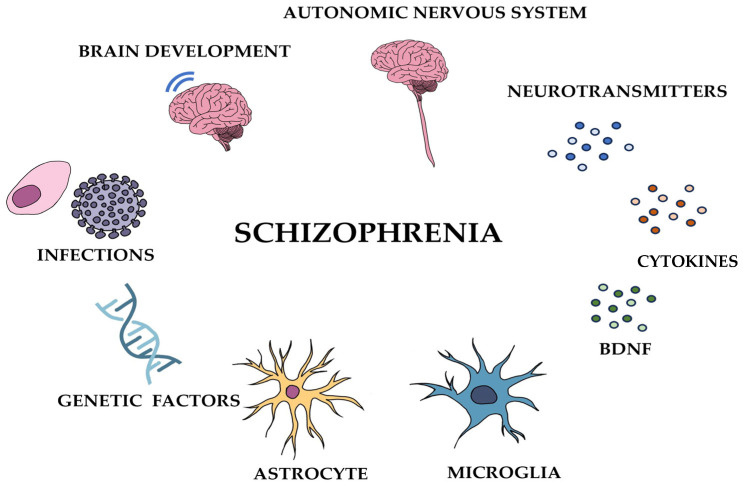
Various factors that can trigger schizophrenia.

Schizophrenia is a severe psychiatric condition that results in a combination of hallucinations, delusions, and profoundly chaotic cognitive processes and behavior [1]. Schizophrenia is a multifactorial disease; its etiology involves a combination of genetic factors, as well as experiences of adversity, infection, and interactions between environmental and genetic influences [2,3,4,5,6,7] (Figure 1). The quantity of individuals diagnosed with schizophrenia witnessed a 65% surge in 2019 compared to the figures from 1990 [8]. Notably, there exists compelling evidence that 85% of individuals diagnosed with schizophrenia experienced childhood trauma [9].

Over the past few decades, the relationship between the gut, microbiota, and the brain has been a subject of significant attention. Extensive experimental evidence highlights the substantial influence that the microbiota can have on both gut and brain functions [10,11]. Communication between the gut and brain occurs in both directions, primarily through various pathways [12]. Recent studies on the gut–brain axis have provided insights into the possible impact of the gut microbiome on the functioning of the central nervous system (CNS) and on mental health conditions such as schizophrenia [13].

The gut microbiota, a diverse community of microorganisms residing in the gastrointestinal tract, plays a crucial role in human physiology and pathology, affecting various biological functions like the maturation of the immune system, the synthesis of essential vitamins, and protection against pathogenic bacteria [14,15,16]. During early life, microbial colonization begins before birth, with delivery mode and environmental factors influencing the composition of the gut microbiota [17,18,19,20]. Disruptions to the gut microbiota, caused by changes in diet, medication use, environmental pollutants, and stress, may lead to imbalances that could have far-reaching effects on CNS function and mental health [21,22].

Especially noteworthy within the context of schizophrenia are short-chain fatty acids (SCFAs), which are metabolic byproducts produced by gut microbes during the fermentation process [23,24,25]. SCFAs have been found to cross the blood–brain barrier and can influence CNS activity, including the modulation of microglia activity and cytokine production [26]. Dysregulation of neurotransmitters like glutamate, γ-aminobutyric acid (GABA), dopamine, and serotonin, produced by gut microbes or their precursor molecules, may also contribute to CNS disorders, including schizophrenia [27,28,29,30,31,32,33,34,35].

The objective of this review is to investigate and elucidate the possible connection involving SCFAs, the gut microbiome, and schizophrenia. By synthesizing the existing literature on this topic, our goal is to deepen our comprehension of the gut–brain axis in schizophrenia and its implications for future research and therapeutic strategies.

## 2. Navigating Schizophrenia

### 2.1. Schizophrenia: Characteristics, Co-Occurring Disorders, and Prodromal Phase

Schizophrenia impacts approximately 1% of the population, usually manifesting during early adulthood, and persisting throughout an individual’s life, with a higher risk observed in males [36,37]. Positive symptoms of schizophrenia include delusions, hallucinations, and disordered thinking, while negative symptoms involve a loss of motivation and emotional expression [38,39]. It is essential to emphasize that not all individuals with schizophrenia are involved in criminal activities. However, in some cases, delusions, hallucinations, or difficulties in social interactions may lead to such acts [40]. Additionally, many individuals with schizophrenia experience co-occurring mental disorders, such as depression and anxiety disorders [41]. Regrettably, the lifetime prevalence of suicide among individuals with schizophrenia is approximately 10% [40]. Impaired cognitive function is a significant feature of schizophrenia [42], and an ongoing debate exists about the nature and progression of cognitive deficits throughout the course of the disorder [43]. Furthermore, schizophrenia often occurs after a prodromal phase, which includes attenuated positive symptoms, mood symptoms, and cognitive deficits [44]. However, these prodromal symptoms are often underestimated by both the individuals experiencing them and society, leading to a lack of recognition that they may serve as potential warning signs [45].

### 2.2. Understanding Schizophrenia: Multifaceted Insights

#### 2.2.1. Etiology of Schizophrenia: Genetic and Environmental Factors

Schizophrenia is closely correlated with genetic factors, particularly involving specific chromosomal regions such as 22q11–13, 6p, 13q, 8p, and 1q21–22, which have been linked to its pathogenesis [2,3]. However, genetic studies have encountered difficulties because the genes implicated often lack consistent associations and are challenging to reproduce [46]. Even though schizophrenia has a high heritability, about 60% of all schizophrenia patients do not have a familial history of the condition [47]. Childhood trauma experiences have shown a strong association with schizophrenia, especially in individuals who are genetically vulnerable. Moreover, adverse childhood experiences can impact the development of psychiatric disorders in general [48]. Reports suggest that individuals who faced adversity during childhood have a 2.78 times higher likelihood of developing psychosis than those who did not experience such adversity [4]. Moreover, repeated exposure to urbanization-related factors during the developmental period could increase the risk of schizophrenia [49]. Furthermore, there is a connection between a history of autoimmune diseases and childhood infections and an elevated risk of developing schizophrenia [5,6]. Maternal genital or reproductive infections have been found to elevate the risk of schizophrenia in offspring [50]. Furthermore, stress or infection during pregnancy, along with maternal malnutrition, can affect fetal brain development and increase the likelihood of schizophrenia [51].

Indeed, exposure to infectious pathogens and inflammatory stimuli profoundly impacts the brain and behavior [52]. For instance, animal studies show that infection with *Toxoplasma gondii* alters behavior and affects the functioning of neurotransmitters, while in humans, *Toxoplasma gondii* infection is associated with symptoms similar to those observed in patients with schizophrenia [53]. Notably, in patients with schizophrenia, CD8 T cells, which are vital for facilitating long-lasting immunity, have been found to be downregulated [54]. *Toxoplasma gondii* infection induces the production of various cytokines by microglia, astrocytes, and neurons [55]. Additionally, it increases dopamine release, the amount of which correlates with the number of infected cells [56]. Apart from *Toxoplasma gondii*, schizophrenia has also been linked to other infections like *Chlamydophila psittaci*, *Chlamydophila pneumoniae*, *Human Herpesvirus 2*, *Borna Disease Virus*, and *Human Endogenous Retrovirus W.* [57]. Notably, a study found that *Toxoplasma gondii* infection affected the gut microbiome of mice [58], highlighting the intricate interplay between environmental and genetic factors in the development of disorders, emphasizing the significance of considering multiple factors in the pathogenesis of schizophrenia [7].

#### 2.2.2. Neural Connectivity and Brain Abnormalities in Schizophrenia

Schizophrenia is associated with deficits in neural connectivity, that are associated with multiple brain development processes, such as myelination, synaptic pruning, and the development of inhibitory neural networks. Synaptic pruning is a critical process during brain development in which connections between neurons, known as synapses, are modified and optimized. Myelination involves the formation of a protective layer called myelin around nerve fibers or axons. Additionally, the physiological maturation of inhibitory neural networks is essential for proper brain function [39,59,60]. During adolescence, the brain undergoes a reorganization of cortical connections through synaptic pruning. Excessive or insufficient pruning during this period can contribute to the development of schizophrenia [61]. The brain consists of gray matter, including nerve cell bodies, dendrites, local axon branching, glial cells, and blood vessels, as well as white matter, composed mostly of long-distance axon bundles [62]. Throughout development and maturation, the brain experiences changes in both gray and white matter. Disruptions in these changes can lead to difficulties in maintaining proper coordination between different brain regions, potentially contributing to schizophrenia [63]. In healthy individuals, the gray matter-to-white matter ratio tends to decrease with age, whereas in patients with schizophrenia, the white matter volume does not increase. Instead, the gray matter-to-white matter ratio increases with age [64]. Patients with schizophrenia also exhibit a significant decrease in white matter compared to healthy subjects, particularly in regions such as the frontal corona radiata (nerve fiber bundles in the frontal lobe) and corpus callosum (a structure connecting the two brain hemispheres) [65]. Furthermore, a consistent reduction in gray matter has been observed in patients with chronic schizophrenia compared to individuals without the condition, regardless of whether they received treatment or not [66].

#### 2.2.3. Neurotransmitters and Their Role in Schizophrenia

Neurotransmitters are naturally occurring chemicals that facilitate communication between neurons throughout the body and play a vital role in regulating various physiological processes [67]. They are released at the synapse, which is the gap between the presynaptic and postsynaptic membranes. After their release, neurotransmitters can either be broken down by enzymes or reabsorbed into the presynaptic neuron’s terminal through reuptake mechanisms for recycling. When neurotransmitters attach to receptors on the postsynaptic membrane, ligand-gated ion channels can either open (causing an excitatory response) or close (resulting in an inhibitory response). This process regulates the passage of Ca^2+^, Na^+^, K^+^, and Cl^−^ ions [68].

Dopamine is a neurotransmitter involved in regulating motor functions, sensory perception, and reward signaling in the mammalian brain [69]. In patients with schizophrenia, there is subcortical dopamine dysfunction, characterized by elevated dopamine function in the presynaptic region of the associative striatum. Furthermore, individuals with an increased risk of developing schizophrenia demonstrate comparable presynaptic dopamine irregularities in the associative striatum [27].

Norepinephrine, produced in the locus coeruleus, plays a role in various functions in the CNS, including arousal, attention, motivation, reward, learning, and memory regulation [70,71]. Dysregulation in the locus coeruleus–norepinephrine system, caused by stress or genetic vulnerability, is associated with the cognitive symptoms of schizophrenia [28]. Additionally, when administered with alpha-methyl-tyrosine, an inhibitor of the initial enzyme in the catecholamine (dopamine, norepinephrine, and epinephrine) synthesis pathway, it has been shown to decrease symptoms of schizophrenia [72].

Glutamate, an excitatory neurotransmitter, has a vital function in facilitating nervous system plasticity and is capable of causing cell death through a process called “excitotoxicity”. It also plays a significant role in memory storage [73]. The enzyme glutamine synthetase, which is expressed in brain astrocytes, can convert glutamate into glutamine, and from glutamine, phosphate-activated glutaminase in glutamatergic neurons can convert it back to glutamate [74]. NMDA glutamate receptors are heteromeric ion channels that are activated by the binding of d-serine/glycine and glutamate. They are controlled by astrocytes and are crucial for synaptic plasticity, learning, and memory. The glutamate hypothesis suggests that a deficiency in NMDA glutamate receptors may lead to symptoms of schizophrenia [29,75,76,77]. Dysfunctions such as NMDAR deficiency can result in pyramidal neuron hyperactivity and increased presynaptic glutamate release, contributing to the development of schizophrenia [30]. Furthermore, elevated levels of glutamine were detected in individuals with schizophrenia, possibly as a result of increased presynaptic release of glutamate caused by NMDAR dysfunction [31]. Additionally, the excitatory amino acid transporters (EAATs) bind to and absorb glutamate. Decreased expression of EAAT1 and EAAT2 was observed in an elderly group with schizophrenia [78], and EAAT1 knockout mice exhibited positive symptoms of schizophrenia [79].

Moreover, excess serotonin induced by stress in the anterior cingulate cortex and the dorsolateral prefrontal cortex is associated with schizophrenia [32]. Serotonin is a neurotransmitter that regulates neuropsychological processes and neural activity and also influences gastrointestinal functions, such as intestinal motility, bladder control, and cardiovascular function [80]. Tryptophan, the only precursor from which serotonin is synthesized, is crucial not just for emotional regulation, sleep, appetite, and pain but also for the functioning of the colon’s muscles and intestinal secretions [81]. In the context of the kynurenine pathway, which is responsible for the degradation of tryptophan, the average tryptophan catabolism index and kynurenine/tryptophan ratio showed significantly elevated levels of expression in the prefrontal cortex of the group of patients with schizophrenia [82].

Histamine is a neurotransmitter that plays a significant role in immune response, inflammation, wakefulness, appetite control, and cognition, and it is involved in various CNS disorders, including schizophrenia [33,83]. The central histamine activity was detected to be heightened in patients with chronic schizophrenia, and the lack of the histamine receptor gene resulted in the manifestation of negative symptoms of schizophrenia [84,85,86].

GABA is an inhibitory neurotransmitter, and aside from schizophrenia, conditions such as anxiety and mood disorders are linked to reduced GABA levels [87]. Postmortem examinations of schizophrenia patients have revealed deficiencies in GABA, indicating a potential contribution to the pathophysiology of schizophrenia [34].

#### 2.2.4. Autonomic Nervous System and Hormonal Factors in Schizophrenia

The autonomic nervous system (ANS) has a vital role in controlling the homeostasis of various bodily functions, including heart rate, respiratory rate, and digestive function. Studies have indicated that ANS function is slower in patients with schizophrenia compared to healthy individuals [88]. Additionally, patients with schizophrenia exhibit suppressed heart rate variability (HRV), and this reduction in HRV has been shown to negatively impact cognitive function in schizophrenia [89]. In the hypothalamic–pituitary–adrenal (HPA) axis, the pituitary gland releases adrenocorticotropic hormone (ACTH), which then triggers the production and release of glucocorticoids in the adrenal cortex [90]. Glucocorticoids are essential for normal brain development, and any disturbances in their levels, whether they are suppressed or elevated, can have harmful effects on brain development [91,92]. As a result, it has been hypothesized that elevated levels of glucocorticoids may contribute to the development of schizophrenia [93].

#### 2.2.5. Immune System Dysregulation and Cytokine Abnormalities in Schizophrenia

Schizophrenia is linked to compromised immune system functioning, and individuals with schizophrenia have displayed abnormal cytokine levels [94]. Cytokines play a crucial role in regulating cell development, growth, survival, and differentiation through autocrine or peripheral secretion pathways [95]. Systemic inflammation has the ability to penetrate the blood–brain barrier (BBB) and trigger neuroinflammation. Peripheral cytokines generated by inflammation can also influence mood and cognitive function with conditions such as aging and obesity having potential impacts on mood and cognitive processes [96,97]. Peripheral cytokines can signal to neurons, astrocytes (which regulate the formation and development of neural circuits in the CNS), and microglia (playing important roles in inflammatory processes and brain development, plasticity, and cognition) [98,99,100,101]. These cytokine signals are involved in processes like neurogenesis, synapse formation, and plasticity [102]. Increased levels of IL-8, IL-1 beta, and IL-6 in the cerebrospinal fluid have been observed in patients with Schizophrenia Spectrum Disorders (SSDs) in comparison to those without the condition, with IL-6 being higher in SSD patients than in those with chronic schizophrenia [103]. Moreover, decreased the volume of cortical gray matter in individuals with schizophrenia was found to be more pronounced in those with high expression of inflammatory cytokines [104]. Chemokines, a subset of cytokines with chemotactic properties, also have neuroimmunomodulatory effects. Among patients with schizophrenia, higher levels of chemokines have been reported, especially in elderly and chronic patients [105,106]. Additionally, enhancers activated in the B-lymphocyte lineage, which is a tissue with important acquired immune functions, have been found to be associated with schizophrenia [107].

#### 2.2.6. The Role of Astrocytes and Microglia in Schizophrenia: Neurodevelopmental and Neuroinflammatory Perspectives

Astrocytes and microglia have the ability to promote inflammation or reduce it, depending on the type of injury or damage they encounter [108]. After a central nervous system injury, astrocytes and microglia become active. Their classification as neurotoxic (A1/M1) or neuroprotective (A2/M2) depends on their activation. Recent research indicates that both astrocytes and microglia can exhibit varying levels of activation and different phenotypes depending on the circumstances [109]. Furthermore, both astrocytes and microglia engage in phagocytic activities, which involve the removal of nerve debris from damaged tissues [110].

Astrocytes, as CNS tissue cells, have a vital role in the development of neural circuits and synaptic activity [99,111]. In the healthy brain, astrocytes have a crucial role in regulating the brain’s environment, providing energy sources to neurons, overseeing synaptic activity, and managing the balance of fluid, ions, pH levels, and neurotransmitters [112]. When there is neuroinflammation, it leads to the formation of type A1 reactive astrocytes, while in the case of ischemia, type A2 reactive astrocytes are generated. These two types of reactive astrocytes have distinct functions and responses in the brain [113]. Selectively removing astrocytes or altering their numbers can lead to cognitive dysfunction [114]. In individuals with schizophrenia, there are modifications in the density and structure of astrocytes, along with shifts in the expression of several proteins, including glial fibrillary acidic proteins, aquaporin 4, S100β, Glutaminase, thrombospondin-1, and EAAT2 [115,116,117,118]. Furthermore, delayed differentiation and abnormal cell forms have been linked to schizophrenia [119].

Microglia, another type of CNS tissue cell, also play a critical role in CNS diseases [26]. Microglia are brain cells that serve as the primary immune regulators within the central nervous system. They contribute to the preservation of functional neural networks, promote the survival and differentiation of oligodendrocytes, and initiate programmed cell death in neurons and neural progenitors, perform immune surveillance, mediate inflammatory responses, and protect against unwanted substances [120]. Microglia can switch between an M1 pro-inflammatory phenotype and an M2 anti-inflammatory phenotype in reaction to alterations in their local environment [121]. They contribute to neural circuit plasticity by regulating synaptic structure and function, particularly involving glutamatergic and GABAergic neurotransmission [122,123]. Microglia can be activated by stress and may impair working memory [124]. “Priming” is the term used to describe the activation and multiplication of microglia in response to neurodegeneration and the buildup of abnormally misfolded proteins. This priming heightens microglia’s sensitivity to subsequent inflammatory triggers, potentially leading to an excessive inflammatory response [125].

In neuropsychiatric disorders, exposure to traumatic experiences during adolescence in offspring with prenatal experiences can increase vulnerability to stress and raise the risk of neuropsychiatric disorders [126]. Active microglia have been detected in patients with schizophrenia as well [127]. The development and activation of microglia can also be impacted by the gut microbiome [10]. Germ-free mice exhibit an immature microglial innate immune response [128].

#### 2.2.7. Neurotrophic Factors and Schizophrenia: The Role of BDNF

Neurotrophic factors, comprising neurotrophins and neuropeptides, are protein molecules released to control the viability, growth, and normal activities of neurons in both the central and peripheral nervous systems. They have a pivotal function in normal neural activity and are also implicated in responses to trauma, ischemia, and neuroinflammatory reactions [129]. Among these factors, BDNF (Brain-Derived Neurotrophic Factor), a neurotrophin, plays a central role in governing synaptic plasticity and the generation of new neurons in the brain [130]. Variations in neurotrophins, such as BDNF, have been associated with schizophrenia and are believed to be part of the molecular mechanism underlying cognitive dysfunction during neurodevelopmental changes [131]. Individuals with schizophrenia typically exhibit reduced blood levels of BDNF [132]. Furthermore, levels of BDNF have been observed to be reduced in the cortex and hippocampus of germ-free rodents [133]. In rats, the gut microbiome has the potential to impact the expression of brain BDNF through the involvement of gut hormones [134].

## 3. Exploring the Gut Microbiota and Its Multifaceted Impacts

### 3.1. The Gut Microbiota: Impacts on Human Physiology, Immune Function, and Brain Health

The microbiota resides in various regions of the body, including the skin, conjunctiva, oral cavity, airway, vagina, and gastrointestinal tract [135]. Among them, the gut microbiota is a multifaceted and constantly changing community of microorganisms that engage with both the surroundings and the human body [136]. The predominant bacterial phyla inhabiting the intestine consist mainly of *Firmicutes*, which include species like *Lactobacillus*, *Clostridium*, and *Enterococcus*, as well as *Bacteroidetes*, encompassing species like *Bacteroides*. In addition, smaller quantities of *Actinobacteria* (including *Bifidobacteria*), *Proteobacteria* (such as *Escherichia coli*), *Fusobacteria*, *Verrucomicrobia*, and *Cyanobacteria* are also present [137,138,139,140]. Approximately 10^14^ bacteria reside in the human gut, exceeding the number of human cells by a factor of 10 and possessing more than 100 times the amount of genomic content compared to human genomic DNA [141].

The gut microbiota has a crucial function in human physiology and disease, aiding in the formation of gastric mucus and promoting enzymatic activity within the mucous lining to support the digestive system [14]. Some bacteria in the gut microbiota serve protective functions against pathogenic bacteria, acting as a barrier and defending against toxins [15]. Additionally, they contribute to immune system development, synthesize essential vitamins [16]. Moreover, the gut microbiota influences epithelial cell proliferation [142] and insulin resistance [143], fundamentally impacting immune and metabolic functions, and contributing to the regulation of central nervous system homeostasis [144]. Certain miRNAs, which are responsible for regulating gene expression by triggering gene silencing or inhibiting translation, possess the capability to penetrate bacterial cells and regulate their growth and gene expression. Moreover, the gut microbiota can also impact the intestinal expression of miRNAs [145]. Studies have shown that bacterial peptidoglycan derived from the gut microbiota can travel to the brain and activate specific pattern recognition receptors (PRRs) within the innate immune system [146].

### 3.2. Early-Life Microbiota: Influences of Birth Mode and Health on Neonatal Gut Microbiota

Microbiota are already present in the placenta, amniotic fluid, and umbilical cord, indicating that microbial colonization begins even before birth [17,18,19]. However, the neonatal gut microbiota closely resemble the microbiota encountered during childbirth, and the mode of delivery significantly influences the composition of the neonatal gut microbiota [20]. Babies delivered vaginally are colonized by bacteria from the maternal feces and vaginal tract, whereas babies born via caesarean section acquire different bacteria from healthcare workers, the surrounding environment, and medical equipment [147]. Embryologically, infants delivered by caesarean section and those born through natural childbirth show variations in their early-life microbiota, and those with compromised health often have a less diverse microbial population [148].

### 3.3. Influences on Gut Microbiota Composition: Diet, Health, Medications, and Aging

**Figure 2:**
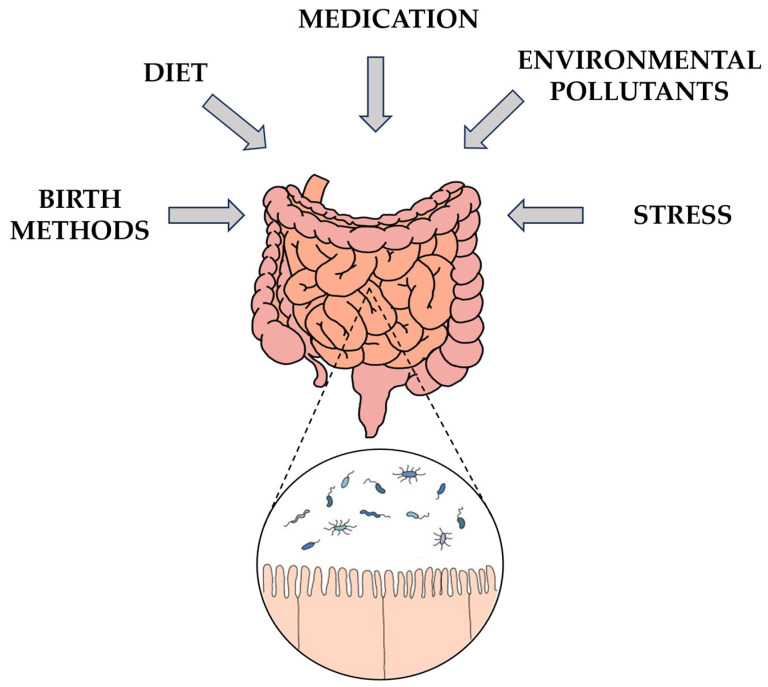
Influences on gut microbiota composition.

The process of gut microbiota colonization undergoes changes based on variations in diet or health conditions [21]. In other words, the intestinal microflora balance is influenced by multiple external factors, including dietary habits, medication usage, environmental pollutants, and stress [22] (Figure 2). Healthy dietary components like a healthful plant-based diet, vegetables, and magnesium are associated with lower levels of pro-inflammatory bacteria, such as *E. coli* and *Clostridium innocuum,* while showing a positive correlation with beneficial anaerobic bacteria like *F. prausnitzii* and *Agathobaculum butyriciproducens* [149]. On the contrary, evidence suggests that the consumption of animal-based foods leads to an elevation in the population of bile-tolerant microorganisms (such as *Alistipes*, *Bilophila*, and *Bacteroides*). Additionally, the genes within these bacteria responsible for encoding microbial bile salt hydrolase, a key enzyme for secondary bile acid production, show notably increased activity when included in an animal-based diet [150]. Substantial rises in bile acids and their metabolites found in fecal matter could have played a role in the disruption of the gut microbiota and may have consequences for the risk of colon cancer [151]. Interestingly, it has been noted that a high intake of saturated fats from animal sources can lead to an elevated presence of *Biophila wadsworthia*, a member of the *Desulfovibrionaceae* family known for triggering acute inflammation through taurine respiration, resulting in the production of hydrogen sulfide. Conversely, when mice were fed unsaturated fats, such as polyunsaturated fats, there was a notable increase in the abundance of beneficial bacteria like *Lactobacillus*, known for its probiotic properties, and *Akkermansia muciniphila*, a species thriving in nutrient-rich environments [152]. A high-fat diet (HFD) induces an imbalance in the gut microbiota and inhibits the metabolism of butyrate, one of the SCFAs produced by the gut microbiota [153]. Furthermore, prolonged deficiency in dietary fiber can result in enduring changes in the composition of the gut microbiome, consequently affecting the development and progression of various diseases [154]. The administration of antibiotics can significantly reduce the richness and diversity of the gut microbiota, leading to reduced levels of serotonin, tryptophan hydroxylase 1, and secondary bile acids in antibiotic-treated rats [155]. The gut microbiota is maintained in a balanced state through enteroendocrine signals and immune responses. When this balance is disrupted, an intestinal bacterial imbalance, also known as “dysbiosis”, occurs, characterized by an increase in specific gut bacteria and elevated enterotoxin levels [156]. This imbalance is associated with an abnormal immune response, leading to the production of inflammatory cytokines [157]. Disruptions in the gut microbiota like these can increase susceptibility to disease [158]. Moreover, the gut microbiota undergoes changes with aging, and there is a significant difference in the microbiota composition between young and elderly adults [159]. A study revealed that certain bacterial species in the aging microbiota promote inflammation when the gut microbiota of an older mouse are transferred to a germ-free young mouse [160].

### 3.4. Intestinal Epithelial Cells and the Microbiota–Gut–Brain Axis: Implications for Immune Function, CNS Disorders, and Overall Health

**Figure 3:**
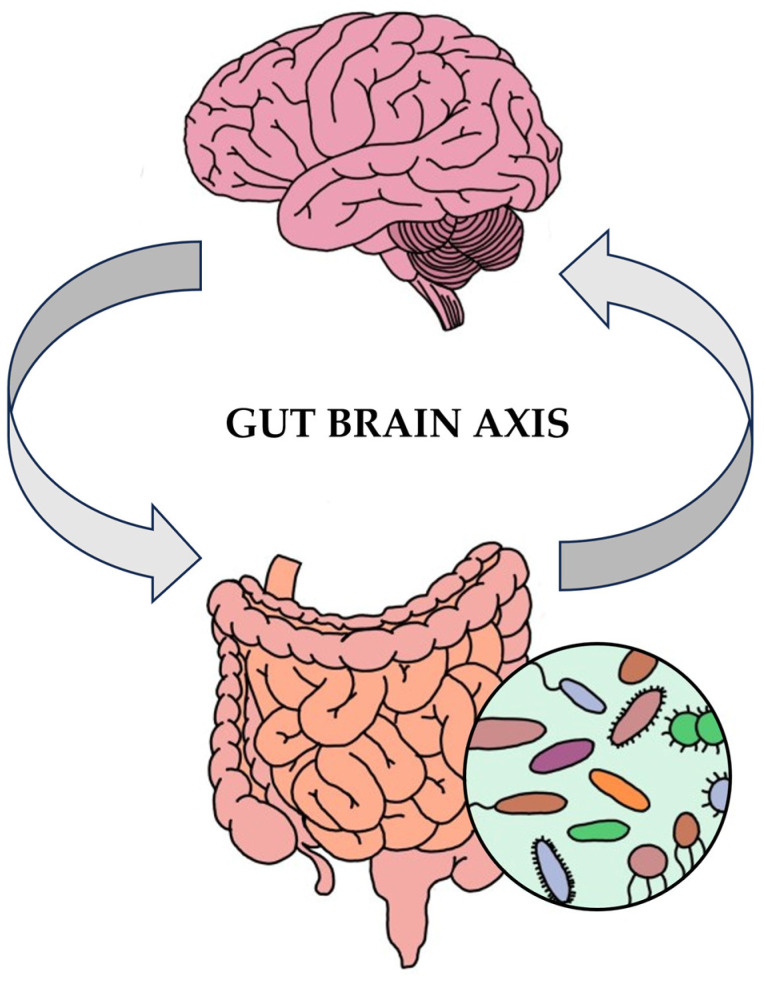
Microbiota–gut–brain axis.

The gut microbiota play a vital role in regulating brain function and behavior through the “microbiota-gut-brain” (MGB) axis [13] (Figure 3). The interaction between the gut and the brain is a two-way one and primarily takes place through several routes, which include the ANS, specifically the enteric nervous system (ENS) and vagus nerve (VN), the HPA axis, the neuroendocrine system, the immune system, and metabolic pathways [12].

The ANS governs involuntary physiological functions such as heart rate, blood pressure, respiration, and digestion. It comprises the sympathetic nervous system (SNS), parasympathetic nervous system (PNS), and ENS [161]. Activation of the SNS triggers the “fight or flight” response, leading to increased heart rate and blood pressure, inhibition of glycogenolysis, and reduced gastrointestinal peristalsis. In contrast, activation of the PNS induces the “rest and digest” response, resulting in a lower heart rate and blood pressure and the resumption of gastrointestinal peristalsis and digestion [162]. The ENS regulates most of the intestinal functions to maintain a healthy microbiota [163]. Neurotransmitters and molecules produce signals that are conveyed to the brain via afferent VN fibers associated with the parasympathetic nervous system (PNS). In turn, the brain sends signals to the enterochromaffin cells (ECCs) and enteroendocrine cells (EECs) within the gut wall, which subsequently engage with the mucosal immune system through efferent VN fibers [164]. Stimulating the VN fortifies the intestinal barrier, lessens inflammation in the peripheral system and restrains the release of pro-inflammatory cytokines [165]. The HPA axis is a physiological stress system responsible for producing glucocorticoids [166]. Glucocorticoids have the potential to impact the brain and behavior, with chronic exposure to high levels associated with depression, while low levels are found among patients with post-traumatic stress disorder [92]. Stress, regulated via the HPA axis, can have consequences on the makeup of the gut microbiota and vice versa [167].

Intestinal epithelial cells (IEC) create a physical barrier separating the intestinal lumen from immune cells, and they fulfill diverse immunological roles. They generate and respond to different cytokines, as well as express molecules that interact with lymphocytes, and have pattern-recognition receptors in their composition [168]. Studies comparing the brains of animals that are colonized (specific pathogen-free, SPF) and those that are germ-free (GF) have shown a significant downregulation of genes related to microglia, indicating that the gut microbiota are crucial for microglia maturation and normalization [128]. The structural elements of bacteria engage with the immune system through Toll-like receptors (TLRs), and gut microbes can activate these receptors as well [169]. In the CNS, astrocytes, microglia, and oligodendrocytes express TLRs, which play roles in innate immunity, CNS autoimmunity, neurodegeneration, and tissue damage [170,171]. Activation of TLRs can induce pro- and anti-inflammatory cytokines [172]. As a result, disruptions or irregularities in the gut–brain axis can result in CNS disorders [173], affecting not only intestinal inflammation, chronic abdominal pain syndrome, and eating disorders, but also neurological conditions including Alzheimer’s disease, Parkinson’s disease, autism spectrum disorder, epilepsy, and major depressive disorder [13,35]. In pivotal studies, male germ-free mice exhibited higher stress responses than normal control rats [133]. Germ-free mice also exhibited heightened neuroendocrine reactions to stress, altered levels of neurotrophins in the hippocampus and amygdala, decreased anxiety and nonspatial memory, and altered brain monoamine neurotransmitter levels [174].

### 3.5. Gut Microbiota Metabolites and Their Impact on CNS Function: Focus on Short-Chain Fatty Acids

The gut microbiota interacts with the CNS not only to regulate food digestion, immune function, and enteroendocrine signal transmission but also through the production of various metabolites, including substances like bile acids, SCFAs, glutamate, norepinephrine, dopamine, GABA, serotonin, and histamine [175]. Among these metabolites, SCFAs have a notable role to play and are generated through the fermentation process of the gut microbiota [25]. SCFAs can pass through the blood–brain barrier (BBB) and engage with microglia, exerting wide-ranging effects on CNS function [26]. SCFAs found in the gut include acetate (C2), propionate (C3), and butyrate (C4) acids, valeric acid (C5), caproic acid (C6), among others, with hydrocarbon tails containing one to six carbon atoms [128,176]. Various bacteria produce different SCFAs. Acetate is generated by bacteria such as *Bifidobacterium bifidum*, *Bifidobacterium infantis*, and *Bifidobacterium breve*. Propionate undergoes three distinct conversions by bacteria. Certain types of bacteria, like *Prevotella* and *Veillonella*, are responsible for converting it into a specific compound called succinic acid. The acrylate pathway is utilized by certain bacterial groups, such as *Coprococcus*, while the propanediol-dependent metabolic pathway is utilized by bacteria like *Roseburia inulinivorans* and *Blautia* species. Butyrate is produced by bacteria from the *Lachnospiraceae* and *Ruminococcaceae* families [177]. These SCFAs activate G protein-coupled receptors (GPCRs), regulating immune responses, anti-inflammatory processes, reactive oxygen species (ROS) induction, and cellular processes [178].

Butyric acid, one of the SCFAs, especially exerts notable influences on the generation of various factors, including BDNF, which promotes the synthesis of neurotransmitters in the CNS through the VN [168]. Additionally, butyric acid, along with acetic acid and propionic acid, has been demonstrated to reduce the expression of genes in the hypothalamus involved in stress signaling, leading to improved behaviors related to stress reactivity and anxiety when administered to mice [179]. Acetate, another SCFA, alters the levels of neurotransmitters like glutamine, glutamate, and GABA in the hypothalamus and increases the expression of neuropeptides associated with anorexia [180]. Furthermore, SCFAs have a wide range of impacts on the immune system. When the ratio of butyric acid to caproic acid increases, the levels of regulatory T lymphocytes rise, while pro-inflammatory T lymphocytes decrease, promoting immune homeostasis [181]. For instance, sodium butyrate inhibits histone acetylation, a process that plays a vital function in aging and memory decline [182]. The gut microbiota also affects the production of serotonin in the colon by influencing intra-intestinal chromaffin cells through the action of SCFAs [183].

SCFAs have been associated with several mental disorders. For example, patients with neuropathy show decreased levels of serotonin, GABA, and dopamine, as well as reduced levels of butyrate, propionate, and acetate [184]. Additionally, the composition of the gut microbiota and SCFA levels differ when comparing individuals with autism to healthy controls. Transplanting the gut microbiota from autistic mice into healthy mice not only leads to autism-like behavior but also results in lower levels of acetate and butyrate and higher levels of valeric acid in the recipient mice [185].

The gut microbiota has a significant role to play in producing and consuming SCFAs and mammalian neurotransmitters, influencing both the CNS and the ENS [186]. However, specific neurotransmitters like GABA, dopamine, glutamate, and serotonin cannot cross the blood–brain barrier directly. Instead, their precursors, which originate from tyrosine and tryptophan, are transported through the blood–brain barrier and then transformed into neurotransmitters within the brain [35]. Of these precursors, tryptophan is predominantly absorbed by the intestine and metabolized by the gut microbiota through three downstream pathways: the 5-hydroxytryptamine pathway, the kynurenine pathway, and the indole pathway [187]. These metabolic pathways produce aryl hydrocarbon receptor (AHR) agonists, which have the ability to limit inflammation in the CNS by influencing astrocytes [188]. Moreover, the absorption of tryptophan in the intestine has regulatory effects on the serotonin and glutamate systems [82].

## 4. Gut Microbiota Dysbiosis in Schizophrenia and Related Disorders

When comparing the metabolic processes involving glucose and lipids of SCFA-producing bacteria and gut microbiota in schizophrenia patients to healthy individuals, SCFA-producing bacteria were less abundant, and the gut microbiota showed abnormal glucose and lipid metabolism [189]. In the comparison between schizophrenia patients and healthy controls, researchers noted a higher abundance of anaerobic bacteria and oral cavity-associated bacteria in the intestines of the patients than in the healthy controls. Remarkably, when researchers transplanted *Streptococcus vestibularis*, an oral bacterium, into mice, it led to the development of schizophrenia-like behavior [190]. Out of the 27 cytokines examined, which included Eotaxin, IL-1β, IL-4, IL-6, IL-8, MIP-1a, and TNF-α, 7 cytokines exhibited significant elevations in schizophrenia patients when compared to healthy individuals. Conversely, in the control group, seven other cytokines, such as IFN-γ, IL-9, IL-1ra, IL-13, MCP-1, MIP-1b, and RANTES, notably decreased in schizophrenia patients. It was noted that schizophrenia patients displayed a negative correlation between the reduced levels of *Faecalibacterium*, *Roseburia*, and *Butyricicoccus*, which play a role in butyrate production, and the aforementioned increased cytokines, while showing a positive correlation with the decreased cytokines mentioned earlier [191]. The changes in the gut microbiota result in the hypoactivity of *N*-methyl-d-aspartate (NMDA) and brain-derived neurotrophic factor (BDNF)/glial-cell derived neurotrophic factor (GDNF) receptors, which regulate brain plasticity, in schizophrenia patients [131,192]. When investigating the connection between the gut microbiota and schizophrenia, researchers compared samples from schizophrenia patients with those from healthy controls. They observed reduced gut microbiota diversity in schizophrenia patients, with 23 operational taxonomic units (OTUs) out of 77 showing increased abundance in the patient group compared to healthy controls. Additionally, when mice were subjected to fecal transplants from individuals with schizophrenia, their neurotransmitter levels in the hippocampus were affected, resulting in reduced levels of glutamate and increased levels of glutamine and GABA [193]. Glutamate plays a crucial role in synaptic plasticity. However, dysfunction in glutamate neurotransmission, particularly disruptions in the signaling of ionotropic glutamate receptors (iGluRs), is associated with schizophrenia and other neurological disorders [194]. Notably, butyrate was also noted for its neuroprotective properties in animal models of Parkinson’s disease, where it reversed reductions in histone acetylation associated with the disease [195,196], and patients with schizophrenia showed a dysregulation of histone deacetylase [196]. Further insights emerged when researchers compared the fecal composition of inpatient schizophrenia patients, distinguishing between those with aggression (ScZ-Ag) and those without (NScZ-Ag). Within the ScZ-Ag group, Prevotella increased, while *Bacteroides*, *Bifidobacterium*, *Faecalibacterium*, *Blautia*, *Collinsella*, and *Eubacterium_coprostanoligenes* decreased. Furthermore, the ScZ-Ag group showed significantly lower levels of acetic acid, propanoic acid, butyric acid, isobutyric acid, isovaleric acid, and isohexanoic acid in their stool compared to the NScZ-Ag group [197]. Moreover, notable changes in the serum concentration of butyric acid were detected in individuals with schizophrenia. Initially, these levels were similar to those in healthy controls, but they increased after treatment [198]. Among the SCFAs produced by gut microbes, valeric acid was found to protect brain cells from excitotoxicity and cell death, while caproic acid, another SCFA, was shown to influence cognitive function, with its levels found to be lower in schizophrenia patients compared to healthy controls [24,199]. In individuals with schizophrenia, the concentration of isovaleric acid was notably elevated compared to healthy controls. Notably, a strong inverse relationship was observed between higher isovaleric acid levels and reduced RBANS scores for both immediate and delayed memory in schizophrenia patients [200]. In patients with schizophrenia, a significant negative correlation was found between the ratio of acetic acid to propionic acid and the sub-scores of working memory and reasoning on the MCCB. This aligns with existing research indicating a connection between SCFA and neurocognitive dysfunction [201].

## 5. Conclusions

In this review, our primary objective was to explore and shed light on the potential relationship between schizophrenia, the gut microbiota, the gut–brain axis, and SCFAs. We investigated whether SCFA, a metabolite produced by the gut microbiota, might be associated with schizophrenia. Our findings suggest that SCFAs can traverse the blood–brain barrier (BBB) and influence CNS activity, including the modulation of microglia activity and cytokine production. Specifically, butyrate appears to impact epigenetic processes, leading to increased histone acetylation.

It is essential to note that there has been limited research into how the gut microbiota influences mental health, particularly in the context of schizophrenia. Most studies have lacked large-scale human clinical trials, and the considerable variability in gut microbiota composition and SCFA production among individuals makes it challenging to establish a clear association with schizophrenia. Additionally, the precise mechanism through which SCFA influences schizophrenia remains unclear, necessitating further research before considering therapeutic applications.

Despite these limitations, our findings hold promise for potential future developments. As the realm of research on the gut–brain axis and its impact on mental health continues to grow, these studies may serve as potential indicators for identifying and predicting of schizophrenia. Additionally, additional progress in this field could result in the creation of innovative treatment approaches that target the gut microbiota for the management of schizophrenia.

In conclusion, while our review highlights the potential link between SCFA, the gut microbiota, and schizophrenia, it is crucial to acknowledge the current limitations and the need for further research. As the scientific community delves deeper into this field, an improved comprehension of the gut–brain axis in schizophrenia could offer valuable insights into diagnosis, treatment, and patient outcomes.

## Data Availability

Not applicable.
